# Whole Blood Transcriptomics Identifies Subclasses of Pediatric Septic Shock

**DOI:** 10.21203/rs.3.rs-3267057/v1

**Published:** 2023-08-28

**Authors:** Jamie O Yang, Matt S. Zinter, Matteo Pellegrini, Man Yee Wong, Kinisha Gala, Daniela Markovic, Brian Nadel, Kerui Peng, Nguyen Do, Serghei Mangul, Vinay M. Nadkarni, Aaron Karlsberg, Dhrithi Deshpande, Manish J. Butte, Lisa Asaro, Michael Agus, Anil Sapru

**Affiliations:** University of California, Los Angeles; University of California, San Francisco; University of California, Los Angeles; University of California, Los Angeles; University of California, Los Angeles; University of California, Los Angeles; University of Southern California; University of Southern California; University of California, Los Angeles; University of Southern California; University of Pennsylvania; University of Southern California; University of Southern California; University of California, Los Angeles; Boston Children’s Hospital; Boston Children’s Hospital; University of California, Los Angeles

**Keywords:** Sepsis, RNA-Seq, Gene Expression, Adaptive immunity, Subclassification

## Abstract

**Background:**

Sepsis is a highly heterogeneous syndrome, that has hindered the development of effective therapies. This has prompted investigators to develop a precision medicine approach aimed at identifying biologically homogenous subgroups of patients with septic shock and critical illnesses. Transcriptomic analysis can identify subclasses derived from differences in underlying pathophysiological processes that may provide the basis for new targeted therapies. The goal of this study was to elucidate pathophysiological pathways and identify pediatric septic shock subclasses based on whole blood RNA expression profiles.

**Methods:**

The subjects were critically ill children with cardiopulmonary failure who were a part of a prospective randomized insulin titration trial to treat hyperglycemia. Genome-wide expression profiling was conducted using RNA-sequencing from whole blood samples obtained from 46 children with septic shock and 52 mechanically ventilated noninfected controls without shock. Patients with septic shock were allocated to subclasses based on hierarchical clustering of gene expression profiles, and we then compared clinical characteristics, plasma inflammatory markers, cell compositions using GEDIT, and immune repertoires using Imrep between the two subclasses.

**Results:**

Patients with septic shock depicted alterations in innate and adaptive immune pathways. Among patients with septic shock, we identified two subtypes based on gene expression patterns. Compared with Subclass 2, Subclass 1 was characterized by upregulation of innate immunity pathways and downregulation of adaptive immunity pathways. Subclass 1 had significantly worse clinical outcomes despite the two classes having similar illness severity on initial clinical presentation. Subclass 1 had elevated levels of plasma inflammatory cytokines and endothelial injury biomarkers and demonstrated decreased percentages of CD4 T cells and B cells, and less diverse T-Cell receptor repertoires.

**Conclusions:**

Two subclasses of pediatric septic shock patients were discovered through genome-wide expression profiling based on whole blood RNA sequencing with major biological and clinical differences.

**Trial Registration:**

This is a secondary analysis of data generated as part of the observational CAF PINT ancillary of the HALF PINT study (NCT01565941). Registered 29 March 2012.

## BACKGROUND

Septic shock, a severe form of sepsis, characterized by profound circulatory and metabolic abnormalities that require the need for a vasopressor to maintain mean arterial pressures, is associated with mortality rates greater than 40%[1]. Despite efforts to develop therapies beyond current standard practices over the past thirty years, over 60 large sepsis clinical trials have failed to identify significant positive results [2]. Many experts now believe that the failure is largely due to the heterogeneity of sepsis patients [3]. This has prompted investigators to develop a precision medicine approach aimed at identifying biologically homogenous subgroups of patients with septic shock and critical illnesses [4–6]. The hope is to develop targeted therapeutic interventions for sepsis patients based on pathophysiological processes and clinical phenotypes rather than a one-size-fits-all-approach. [7–11].

Wong et al., pioneers in the field, were the first to delineate septic shock subgroups among pediatric patients, based on underlying biological perturbations and establish a potentially prognostic and predictive sepsis subclassification system. Their work was based on microarray-based gene expression data, discovering distinct subclasses characterized by differential gene expression related to adaptive immunity and glucocorticoid receptor signaling. Remarkably, these subclasses, derived independently of clinical data, exhibited variations in mortality rates and responses to physician-prescribed corticosteroids, highlighting their clinical significance However, differences if any, in circulating protein biomarkers and immune cell repertoires between these subclasses are not known [5].

Although microarray platforms have traditionally served as established and reliable tools for gene expression profiling, the emergence of RNA sequencing (RNA-seq) introduces a potent technology capable of directly sequencing amplified cDNA to measure transcript abundance. This relatively recent approach enables sensitive transcript detection and encompasses a broader quantitative spectrum of expression level changes compared to microarrays. Consequently, it yields more accurate estimations of absolute transcript levels, identifies a greater number of differentially expressed protein-coding genes, and exhibits enhanced concordance between RNA-Seq and protein expression measurements. Moreover, RNA-Seq holds the potential for comprehensive biological exploration such as cell deconvolution and imputation of B and T Cell repertoires surpassing the capabilities of microarrays [12–14].

In this study, we leveraged available RNA-seq data from a clinical trial of children with circulatory and/or respiratory failure and compared whole genome expression between cases with septic shock and mechanically ventilated controls. Next, we used an unsupervised discovery-based approach to identify two pediatric septic shock subclasses based on their transcriptomic signatures and compared their clinical characteristics, ionotropic use, and circulating biomarkers. Finally, to further expand on the knowledge of biological differences between the subclasses, we imputed immune cell abundance, and T and B-cell repertoires from the sequencing data and compared these between the subclasses.

## METHODS

### Aim

The first aim was to elucidate genetic and biological differences in pediatric septic shock patients compared to critically ill controls. The second aim was to identify clinically significant pediatric septic shock subclasses based on whole blood RNA expression profiles and compare clinical characteristics, circulating protein biomarkers, immune cells, and adaptive immune repertoires.

### Design, Setting, Patient Characteristics

**Figure 1:**
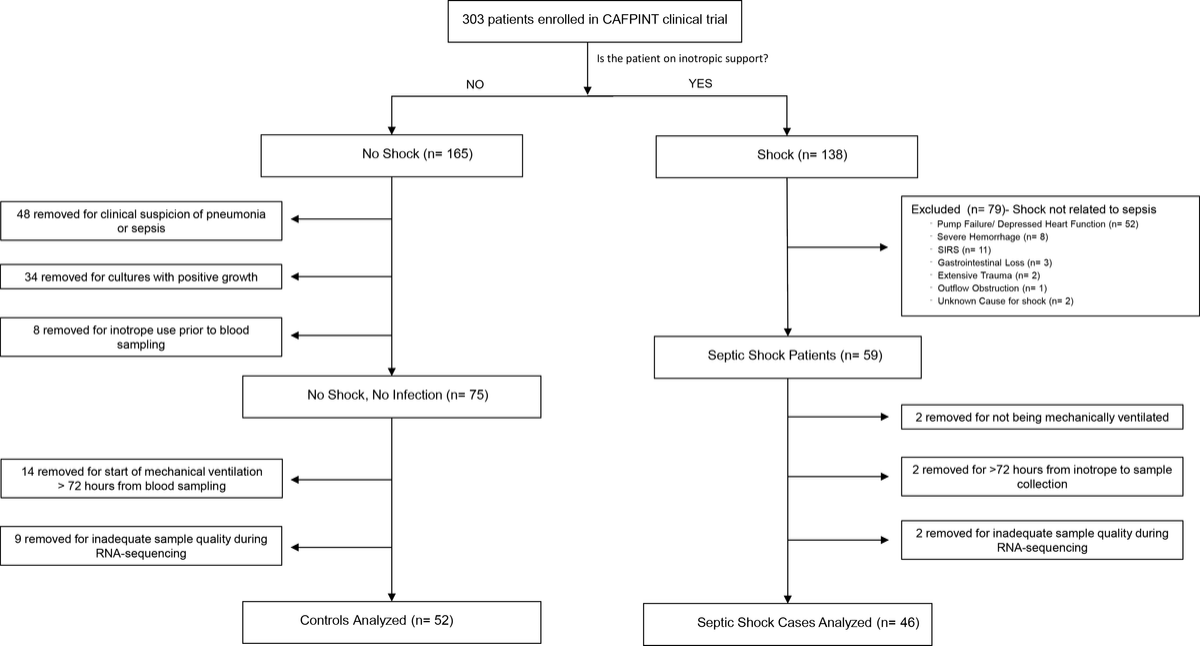
Flow chart of patients in this study after applying exclusion and inclusion criteria. All septic shock cases were diagnosed with sepsis and were started on inotropes <72 hours from blood sampling. All controls had no documented diagnosis of sepsis or pneumonia, no positive cultures, and no inotrope use, and were started on mechanical ventilation <72 hours from blood sampling.

This was a nested case-control study. Subjects included in this study were a subset of the patients from the Coagulation and Fibrinolysis in Pediatric Insulin Titration Trial (CAF-PINT) [11] an ancillary to the Heart and Lung Failure Pediatric Insulin Titration Trial (HALF PINT) (ref 23). All were mechanically ventilated, had blood glucose levels greater than 150 mg per deciliter and were treated with continuous insulin infusion randomized to one of two targeted glycemic control ranges. Blood samples were collected prior to initiation of the randomized intervention. Septic shock cases were defined as patients who had a documented clinical diagnosis of sepsis and were started on inotropes within 72 hours prior to blood sampling. Controls were defined as patients who had no documentation of infection or sepsis, no positive cultures and did not require inotropic support prior to randomization. (Fig. 1, Table 1).

### RNA isolation, sequencing, and expression quantification

Total RNA was extracted from whole blood using the PAXgene Blood RNA Kit modified for pediatric use. Next, sequencing libraries were prepared using the Nugen universal plus kit with polyA capture and sequenced with the NovaSeq S4 system (Illumina) to generate 2×150 base paired-end reads to a target depth of 50 million read-pairs per sample. After quality control, 20,010 protein-coding genes were left for analysis. Additional details on these methods are found in the Online Data Supplement.

### Differential gene expression analysis

For Aim 1, we compared baseline gene expression between cases and controls using negative binomial generalized linear models (EdgeR). Differentially expressed genes (DEGs) were defined as genes with an absolute log_2_-fold change of greater than 0.5 and a false discovery rate (FDR) of less than 0.05. Then, we used EnrichR, a pathway enrichment tool, to identify significant GO terms and KEGG pathways in our list of DEGs [15]. Terms and pathways with adjusted p values less than 0.05 were retained. For Aim 2, patient values for these statistically significant DEGs underwent variance-stabilizing transformation and were plotted in a heatmap with unsupervised hierarchical clustering based on Spearman correlation. Unsupervised hierarchical clustering is a technique to group individuals into clusters based on similarities, with the distance or difference between individuals represented by a dendrogram. As was previously done by Wong et al [16], two septic shock subclasses were ultimately chosen based on first-order dendrogram branching (Fig. 3A), as Subclass 2 patients appeared visually more similar to controls, and Subclass 1 was clustered separately in a different clade. Clinical features, circulating biomarkers, immune cells and adaptive immune repertoires were then contrasted using subclass membership.

### Comparing Clinical Characteristics

We compared the clinical outcomes between cases and controls and then between the two subclasses using nonparametric linear regression analysis with bootstrapping, adjusting for baseline differences between treatment groups including PRISM score and steroid use.

### Biomarker quantification

Plasma levels of twelve biomarkers of inflammation and endothelial injury were assayed using the Human Magnetic Luminex Screening Assay with the Luminex 200 (R&D Systems) according to the manufacturer’s instructions at the UCLA Immune Assessment Core facility. Values were compared across septic shock subclasses using the Wilcoxon rank-sum test and the false discovery rate criterion to adjust for multiple testing.

### Measuring Whole Blood Cell Type Composition

We applied the Gene Expression Deconvolution Interactive Tool (GEDIT), a deconvolution tool that utilizes bulk gene expression data to model the most likely combination of cells that would produce the presented bulk transcriptome to estimate cell type fractional composition within whole blood [17, 18]. The abundance of each cell type in each subclass was then compared using a Wilcoxon rank-sum test, followed by a Bonferroni correction.

### Profiling of Adaptive Immune Repertoires

We then sought to quantify adaptive immune responses based on the recombination landscape of genes encoding B and T-cell receptors (BCR and TCR). We utilized ImReP, a computational method for profiling the adaptive immune repertoire from RNA-seq data [19]. Subsequently, a comparison between the two subgroups was performed using a Wilcoxon rank-sum test on the number of reads associated with each receptor type.

## RESULTS

### Patients

Of 303 CAF-PINT patients, we identified n = 46 with septic shock and n = 52 controls without sepsis or shock (Fig. 1). Septic shock patients were older, whereas controls were younger and more likely to be admitted for asthma, bronchiolitis, postoperative care, or trauma (Table 1).

### Identification of septic shock subclasses

**Figure 2:**
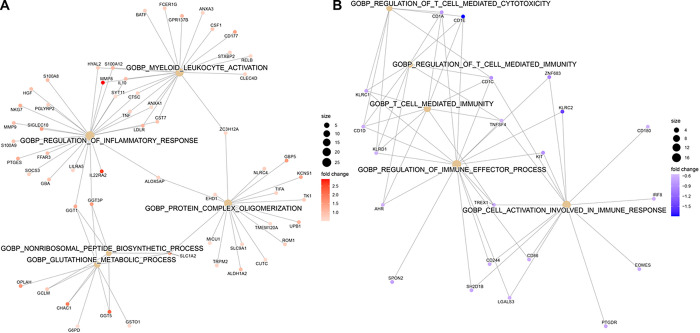
Gene network displaying functional enriched terms with associated DEGs between septic shock cases and controls A. Upregulated pathways in septic shock cases compared to controls B. Downregulated pathways in septic shock cases compared to controls

**Figure 3:**
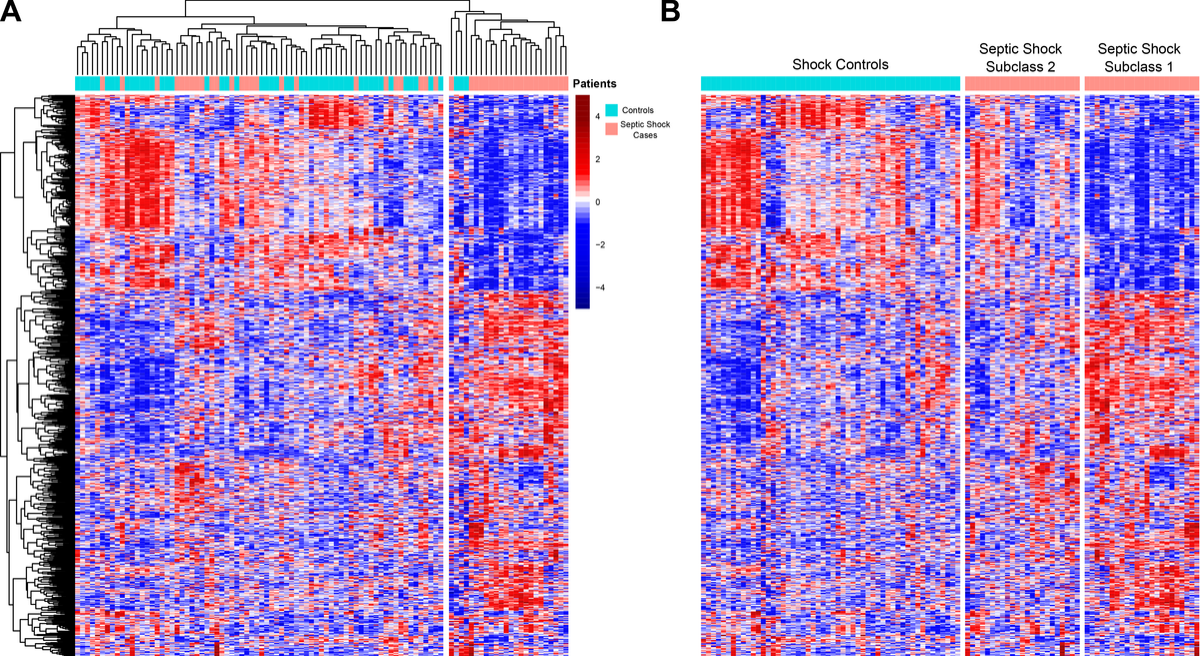
Heatmaps A. Heatmap depicting the expression of all 840 differentially expressed genes (DEGs) between septic shock cases and controls. Each row represents a DEG; blue indicates that a gene is downregulated, while red indicates that a gene is upregulated. Each column is a patient, so each column depicts the transcriptomic signature of each patient. The dendrogram at the top shows unsupervised hierarchical clustering of the patients such that the patients with similar transcriptomic signatures are clustered together. B. This heatmap depicts the same patients and genes as in Figure 2A, but now the controls are sorted on the left, and the cases sorted on the right. Clustering based on each patient’s transcriptomic signature was performed within each group.

We first identified genes that were differentially expressed between septic shock cases and controls. A total of 840 DEGs were identified: 530 were upregulated and 310 were downregulated in patients with septic shock compared to controls (FDR < 0.1, **Table E7**). Pathway enrichment analysis using EnrichR showed that septic shock patients had increased expression of pathways related to neutrophil degranulation and glutathione metabolism and decreased expression of pathways related to antigen processing and presentation and T cell activity (Fig. 2, **Table E1**). The 840 genes were subjected to unsupervised hierarchical clustering based on a correlation distance matrix as shown in the heatmap in Fig. 3A. Overall, septic shock cases appear clustered to the right, and critically ill controls are clustered to the left. Interestingly, there was heterogeneity among the septic shock cases. The transcriptomic signature of cases on the right appears significantly different from the septic shock cases on the left, which are more similar to controls. Based on first-order branching of the dendrogram of the columns, two major septic shock subclasses were identified. We designated these subclasses arbitrarily as Subclass 1 and 2 (Fig. 3B).

### Differing Clinical Characteristics Between the Septic Shock Subclasses

Given the biological differences between the two subclasses, we investigated differences in clinical outcomes. We first compared baseline characteristics between the two groups, and the demographic and clinical data are shown in Table 1. We found that Subclass 1 patients were on average significantly older and had higher average blood glucose levels at baseline. There was no difference between subgroups in ICU admission illness severity, as calculated by the Pediatric Risk of Mortality (PRISM) score. Despite the absence of a difference in mortality risk, these two subgroups had significantly different clinical outcomes (Table 2). Over the course of the study, Subclass 1 patients had statistically higher maximum Pediatric Logistic Organ Dysfunction (PELOD) scores indicating more severe multiple organ dysfunction. Subclass 1 also had higher maximum Vasoactive-Inotropes Scores, indicating that they required more cardiovascular support. Subclass 1 also had fewer hospital-free days and longer ICU stays than Subclass There were no statistically significant differences in ventilator-free days. No differences in mortality were seen, as only three patients total died after 90 days of follow up.

### Biomarker analysis

Given the finding of greater innate and myeloid-derived inflammation as well greater circulating endothelial cells among patients in Subclass 1, we next tested whether gene expression findings could be recapitulated using plasma protein markers. We examined a panel of twelve candidate inflammatory biomarkers of sepsis to identify differences between the two subclasses. These markers were initially chosen to provide insights into derangements of inflammation and thrombosis pathways based on their association with hyperglycemia and pediatric critical illness [20–22]. Subclass 1 had significantly higher levels of PAI1, IL6, IL8, IL10, ANG2, TREM1, and TNFR than Subclass 2 (Table 3). There were no significant differences between subclasses of IL4, thrombomodulin, TFPI, P-selectin, and ICAM1. In comparing each subclass individually to controls, Subclass 1 had significantly higher levels of PAI1, IL6, IL8, IL10, ANG2, TREM1, and TNFR-1; while Subclass 2 and controls did not have many significant differences relative to non-septic critically ill controls **(Table E6).**

### Differential gene expression across septic shock subclasses

**Figure 4:**
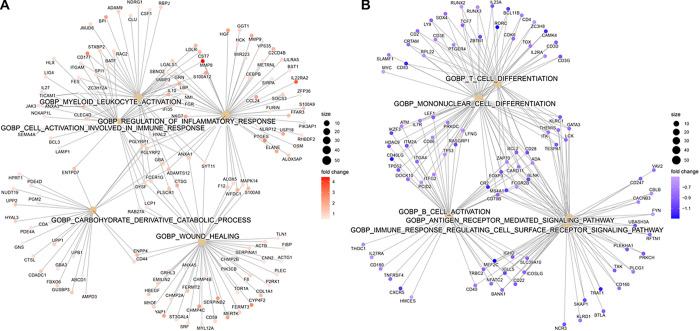
Gene network displaying functionally enriched terms with associated DEGs between Subclass 1 and Subclass 2. A. Upregulated Pathways in Subclass 1 compared to Subclass 2 B. Downregulated Pathways in Subclass 1 compared to Subclass 2

Using these two septic shock subclasses, Subclass 1 (n = 21) and Subclass 2 (n = 25), as comparison groups, we performed differential gene expression analysis and found a total of 2486 DEGs **(Table E8).** A total of 1275 of these genes were upregulated and 1211 were downregulated in Subclass 1 compared to Subclass 2 (FDR < 0.05). Pathway enrichment analysis of the 2486 genes revealed upregulation in neutrophil-mediated immunity and downregulation in adaptive immunity pathways including B and T-cell activity, in Subclass 1 compared to Subclass 2 (Fig. 4, **Table E2**).

### Cell Deconvolution Analyses and Profiling of Adaptive Immune Repertoires

**Figure 5:**
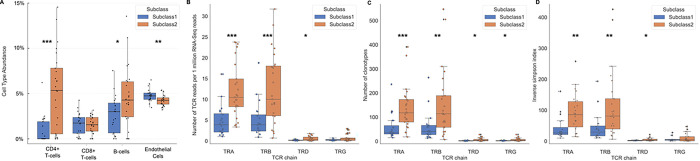
A. Box plot depicting differences in whole blood cell type abundances between Subclass 1 and 2. Subclass 1 has significantly lower percentages of CD4 T cells (0.9 ± 1.5 vs 5.2 ± 4.3; p<0.001) and B cells (2.7 ± 2.0 vs 4.6 ± 3.3; p = 0.024), and a higher percentage of endothelial cells compared to Subclass 2 (4.8 ± 0.7 vs 4.2 ± 0.6). There were no significant differences in percentages of CD8 T cells between the two subclasses. B. Number of TCR reads per 1 million RNA-Seq reads in septic shock subclasses. Subclass 1 had significantly fewer TCR reads normalized per RNA-sequencing reads than Subclass 2 (3.91 ± 4.30 vs 10/03 ± 8.46; p = 0.001). C. Subclass 1 had significantly fewer mean clonotypes than Subclass 2 (59.70 ± 59.49 vs 155.96 ± 138.4; p = 0.002). D. Inverse Simpson index, a measure of mean diversity of the T-cell repertoire, between Subclass 1 versus Subclass 2. Subclass 1 had a less diverse mean T-cell repertoire than Subclass 2 on the Inverse Simpson index (43.84 ± 45.11 vs 114.45 ± 108.8; p = 0.002)

To quantify cell type populations in our samples, we performed expression-based cell deconvolution analysis [17]. In concordance with our pathway analysis, we found that compared to Subclass 2, Subclass 1 had significantly lower percentages of CD4 + T cells (0.9 ± 1.5 vs 5.2 ± 4.3; p < 0.001), B cells (2.7 ± 2.0 vs 4.6 ± 3.3; p = 0.024), and dendritic cells (3.0 ± 0.9 vs 3.8 ± 1.0, p = 0.007), and a higher percentage of endothelial cells (4.8 ± 0.7 vs 4.2 ± 0.6, p = 0.004) and adipocyte cells (2.6 ± 0.5 vs 2.4 ± 0.5; p = 0.005) (Fig. 5A, **Table E3**). There were no significant fractional differences in other cell types, including CD8 + T cells or neutrophils, between the two subclasses (**Table E3**). We considered that in addition to a decrease in T-cell populations, there may also be differences in the T-cell repertoire between the subclasses, which can be a surrogate for monitoring the effectiveness of the adaptive immune system. Using ImReP, we found that compared to Subclass 2, Subclass 1 had significantly fewer T-cell clonotypes (Fig. 5B, **Table E4)** and lower activities and diversity of TCRA, TCRB, and TCRD. (Fig. 5C,D, **Table E5).**

## DISCUSSION

We provide important insights into the pathophysiology of septic shock and the heterogeneity of biological perturbations within patients with septic shock. We compared the expression profiles of children with septic shock to mechanically ventilated controls and found upregulated expression of innate immune and neutrophil pathways and downregulation of adaptive immune pathways in children with septic shock. On further analysis, the expression profiles of patients with septic shock clustered around two subclasses with differential upregulation of innate immunity and downregulation of adaptive immunity. While both subclasses were clinically identified as “septic shock,” Subclass 1 was characterized by upregulation of innate immunity and biomarker profiles consistent with a hyperimmune response along with concomitant downregulation and exhaustion of both B and T cells and lesser T-cell receptor diversity. This same subclass was associated with worse clinical outcomes, underscoring the importance of addressing sepsis heterogeneity. Subclass 2, while having the same clinical diagnosis, appeared biologically much more similar to our critically ill controls.

Our study suggests that patients with severe initial perturbations of the innate and adaptive immune systems, such as those in Subclass 1, are likely to have worse outcomes including higher PELOD scores, higher maximum inotrope usage, and longer intensive care unit and hospital stays, despite the lack of significant differences in illness severity or the Pediatric Risk of Mortality (PRISM) score on initial presentation, indicating that conventional clinical scores alone are often are not sufficient to capture the pathophysiological complexity or outcomes of septic shock. An interesting clinical difference in our cohort is that Subclass 1 patients tended to be older and had modestly higher average blood glucose levels at baseline. [23]. IL-6 is known to contribute to hyperglycemia through insulin resistance, and multiple retrospective analyses have reported that hyperglycemia is associated with adverse outcomes [24].

Not only did Subclass 1 have upregulated genes related to neutrophil immunity compared to Subclass 2, but these patients also had significantly higher levels of proinflammatory cytokines (PAI1, IL6, IL8, ANG2, TREM1, and TNFR1), and a greater degree of organ dysfunction. Early unchecked innate immune-driven inflammation has been associated with a more profound degree of organ injury. Concomitantly, sepsis has also been shown to cause adaptive immunosuppression with a marked loss of T and B cells [25]. Subclass 1 had significant downregulation of genes related to T and B-cell activity, and a lower percentage of CD4 T cells and B cells compared to Subclass 2. Severe T-cell dysfunction, leading to decreased T-cell cytotoxicity and T-cell apoptosis in the setting of a high antigen load and elevated cytokines, is known to occur in septic shock [25].It is a source of debate whether it is the innate immune-driven hyperinflammation, adaptive immunosuppression, or a contribution of both is the driver of morbidity and mortality in sepsis. A recent study supported the latter, as they demonstrated that during sepsis, the proliferation of a large population of immature neutrophils inhibited the proliferation and activation of CD4 + T cells, and a subset of patients with higher frequencies of the immature neutrophils had poorer outcomes [26]. A potential consideration here is that the different sepsis subclasses could represent different chronological stages of the same underlying pathobiology. We attempted to mitigate this confounder by excluding patients who initiated vasoactive support > 72 hours prior to enrollment.

### Comparison to Sepsis Subclasses in the Literature

In the adult population, at least five independent research groups have identified sepsis subgroups describing an adaptive immunity suppression phenotype with corresponding higher mortality such as Scicluna et al.’s MARS1 endotype [27], Davenport et. al.’s SRS1 subgroup [28], and Sweeney et al.’s Inflammopathic subtype [29–31].

In the pediatric population, Hector Wong et. al. were the first to identify two pediatric sepsis endotypes, A and B. Endotype A was characterized by upregulation of innate immunity pathways and repression of pathways related to the adaptive immune system and glucocorticoid receptor signaling. However, the gene expression pattern that differentiates adult SRS groups was not enriched in the pediatric endotypes [32, 33]. This suggests that there may be differences between adults and children, highlighting the importance of studying sepsis specifically in the pediatric population.

Our findings and subclassification are consistent with previously published subclasses in the pediatric sepsis population. Our Subclass 1 was analogous to the Wong et al. pediatric Endotype A, which was identified in a separate pediatric cohort. Similar to Endotype A in Dr Wong’s cohort, Subclass 1 was also characterized by repression of the adaptive immune system related to T-cell activation and had worse clinical outcomes than Subclass 2. We compared our gene list with the previously validated 100 gene signature that differentiated Endotype A and B, and found 12 of the 100 genes present in our list (GNAI3, PLCG1, CD3E, CD247, NCR3, ARPC5, ZAP70, FYN, SEMA6B, TLR8, CAMK2D, TLR8) [34]. In 2021, Muszynski et al. also identified a subclass of septic shock children with immunoparalysis with worse clinical outcomes [35]. They found that this subclass’s transcriptomic profiles demonstrated upregulated pathways in leukocyte extravasation, and downregulation in adaptive immunity pathways [35]. When comparing our Subclass 1 DEGs to Musznski’s immunoparalysis subclass, shared upregulated DEGs included COL17A1, LAMA2, FFAR3, RAP1GAP, XCR1, MMP27, and MMP8, and shared downregulated DEGs included KLRC1 and IL2RB. Thus, while Wong’s Endotype A, Muszynski’s immunoparalysis subclass, and our Subclass 1 are similar, challenges remain in identifying an appropriate panel of candidate genes out of the often large lists of DEGs that can be generalized across the pediatric population. More research in different pediatric cohorts is needed to develop a consensus subclassification system.

At the molecular level, T-cell diversity plays a key role in the ability of the adaptive immune system to effectively mount a response to invading pathogens. Each TCR is made up of alpha (TCRA) and beta chains (TCRB) or delta (TCRD) and gamma (TCRG) chains. TCR diversity is generated through V(D)J recombination in the early stages of T-cell maturation in the thymus, and is critical to effectively recognizing antigen peptides. In the adult sepsis population, studies have shown that septic patients present with a marked decrease in TCR diversity after the onset of shock, which is associated with mortality and the development of nosocomial infections [36, 37]. However, there is a paucity of literature looking at TCR diversity in pediatric sepsis, and one study demonstrated that the TCR repertoires in adults and children are discrepant and thus difficult to directly compare [38]. Our study demonstrated that Subclass 1 patients demonstrated reduced TCR diversity and clonality, including decreased diversity of TCRA, TCRB, and TCRD, with associated worse clinical outcomes at the onset of septic shock.

The role of B cells in sepsis is less understood, but previous studies in the adult population have demonstrated a decrease in B-cell numbers in sepsis. Similar to T-cell immunosuppression, B cells may be shifted toward an exhausted profile during septic shock, leading to increased IL-10 production and depletion of B cells, and worse outcomes. Subclass 1 had significantly higher levels of IL-10 as well as decreased levels of B cells in cell deconvolution analyses compared to Subclass 2.

Our study is novel, as for the first time it not only provides a comprehensive evaluation of dysregulated pathways based on genome-wide differential gene expression but also enhances it with cell deconvolution and T and B-cell receptor diversity estimations in the context of plasma biomarkers and clinical characteristics in septic shock to better characterize and subphenotype the biological perturbations in septic shock that are biologically plausible and clinically relevant.

### Limitations of the Study

First, given that this was a retrospective analysis of a prospective pediatric clinical trial, a limitation was that a suitable case and control population were identified post-retrospectively. We assumed accurate and timely documentation of sepsis and initiation of inotropes for cases, and clinician-documented reasons for PICU admission and initiation of mechanical ventilation and negative blood cultures to create a control group. However even in the unlikely scenario that an underlying infection was missed in our controls, our cases required inotropes, indicating that on the clinical spectrum, sepsis in our cases was certainly much more severe. The use of mechanically ventilated controls ensured that our results were specific to septic shock and not just a result of generalized critical illness. Another limitation was that we could not differentiate between the different sources of sepsis each patient may have had. Finally, our sample sizes were relatively small. A larger sample size would have increased statistical power to detect clinically meaningful differences within each subclass; therefore, mortality was not a reported outcome in this study given that only three of the patients died within our cohort. Therefore, while Subclass 1 had worse outcomes, we cannot state that they had higher mortality.

## CONCLUSIONS

We identified two subclasses of children with septic shock based on differential gene expression using RNA-seq. These two subclasses have differential regulation of genes related to the immune system that is relevant to the pathophysiology of sepsis and septic shock. One subclass is characterized by upregulation of innate immunity pathways and repression of adaptive immunity pathways, with lower levels of T cells and B cells. This subclass is associated with clinically worse outcomes. Thus, subclassifying patients with septic shock based on genome-wide expression data can aid in identifying both new targets for therapies and which patients would likely benefit from them.

## Figures and Tables

**Table 1:** Baseline Characteristics of Septic Shock Cases and Controls

Measures		Controls n (%)	Septic Shock Cases n (%)	P Value†
		N = 52	N = 46	
**Demographics**				
Age-years ‡		5.5 ±4.9	9.0 ±5.6	0.001*
Male §		31 (59.6%)	22 (47.8%)	0.31
Race §	White	34 (65.4%)	30 (65.2%)	0.18
	Black	15 (28.8%)	9 (19.6%)	
	Asian	0 (0.0%)	4 (8.7%)	
	American Indian	0 (0.0%)	1 (2.2%)	
	Mixed	1 (1.9%)	1 (2.2%)	
	Declined or Unknown	2 (3.8%)	1 (2.2%)	
Clinical Characteristics				
	PRISM Score ‡	9.1 ±7.8	13.6 ± 8.5	0.005*
	Average Blood Glucose ‡	122.9 ± 30.7	132.0 ± 34.8	0.13
	Steroid Use §	36 (69.2%)	37 (80.4%)	0.20
**Primary Reason for Admission to Intensive Care Unit** §				< 0.001*
	Cardiovascular (including shock)	1 (1.9%)	11 (23.9%)	
	Respiratory	26 (50.0%)	27 (58.7%)	
	Following elective procedure	7 (13.5%)	2 (4.3%)	
	Following Emergent Procedure	1 (1.9%)	2 (4.3%)	
	Gastrointestinal or Liver	0 (0.0%)	3 (6.5%)	
	Metabolic	1 (1.9%)	0 (0.0%)	
	Neurologic	8 (15.4%)	1 (2.2%)	
	Oncologic	1 (1.9%)	0 (0.0%)	
	Trauma	7 (13.5%)	0 (0.0%)	
**Reason for Initiating Mechanical Ventilation** §				< 0.001*
	Acute respiratory failure related to sepsis	0 (0.0%)	23 (50.0%)	
	Aspiration pneumonia	1 (1.9%)	1 (2.2%)	
	Asthma or reactive airway disease	8 (15.4%)	3 (6.5%)	
	Bronchiolitis	8 (15.4%)	2 (4.3%)	
	Laryngotracheobronchitis	2 (3.8%)	0 (0.0%)	
	Neurologic	11 (21.2%)	2 (4.3%)	
	Pneumonia/hypoxia	0 (0.0%)	11 (23.9%)	
	Procedural	17 (32.7%)	4 (8.7%)	
	Pulmonary Edema	2 (3.8%)	0 (0.0%)	
	Thoracic Trauma	3 (5.8%)	0 (0.0%)	
**Clinical Trial Allocation (two groups)** §				
	Group 1 (lower target blood glucose 80—110 mg/dL)	23 (44.2%)	18 (39.1%)	0.61
	Group 2 (higher target blood glucose 150–180 mg/dL)	29 (55.8%)	28 (69.1%)	0.61

* Significant p values with FDR < 10% are noted with an asterisk.

† P-value was computed using chi-square test for categorical variables, and the Wilcoxon rank sum test for continuous variables

‡ Data presented as mean +/- standard deviation

§ Data presented as n (percentage)

**Table 2:** Clinical Characteristics and Outcomes of Subclass 1 and Subclass 2

Measures		Subclass 1	Subclass 2	P Value
		N = 21	N = 25	
Demographics				
Age-years ‡		11.4 ± 5.3	7.0 ±5.1	0.004*
Male**		9 (42.9%)	13 (52.0%)	0.54
Race**	White	15 (71.4%)	15 (60%)	0.23
	Black	2 (9.5%)	7 (28%)	
	Asian	3 (14.3%)	1 (4%)	
	American Indian	1 (4.8%)	0 (0%)	
	Mixed	0 (0%)	1 (4%)	
	Declined or Unknown	0 (0%)	1 (4%)	
**Baseline Clinical Characteristics**				
	PRISM Score ‡	15.1 ± 7.5	12.4 ± 9.3	0.25
	Average blood glucose (mg/dl) ||	134 (123–170)	116 (105–125)	0.021*
	Clinical Trial Allocation (two groups total, n assigned to group 2)**	15 (71.4%)	13 (52.0%)	0.17
**Baseline Primary Reasons for Admission** **				0.12
	Cardiovascular (including shock)	5 (23.8%)	6 (24.0%)	
	Following elective procedure	0 (0%)	2 (8.0%)	
	Following emergent procedure	2 (9.5%)	0 (0%)	
	Gastrointestinal or Liver	3 (14.3%)	0 (0%)	
	Neurologic	0 (0%)	1 (4.0%)	
	Respiratory (including infections)	11 (52.4%)	16 (64.0%)	
**Baseline Primary Reasons for Mechanical Ventilation** **				0.78
	Acute respiratory failure related to sepsis	13 (61.9%)	10 (40.0%)	
	Aspiration pneumonia	0 (0%)	1 (4.0%)	
	Asthma or reactive airway disease	1 (4.8%)	2 (8.0%)	
	Bronchiolitis	1 (4.8%)	1 (8.0%)	
	Neurologic	1 (4.8%)	1 (4.0%)	
	Pneumonia/hypoxia	4 (19.0%)	7 (28.0%)	
	Procedural	1 (4.8%)	3 (12.0%)	
**Clinical Outcomes**				**Adjusted p value** †
	Maximum PELOD Score‡	18.8 ± 2.2	13.0 ± 1.4	0.026 *
	Maximum Vasoactive-Inotrope Score‡	21.5 ± 5.0	9.8 ±2.7	0.045*
	ICU Length of Stay from Randomization to Discharge (days) ‡	± 5.0	10.4 ± 2.2	0.021*
	Hospital Free Days through Day 28 (days) ‡	5.7 ±1.5	11.1 ± 1.4	0.011*
	Mortality (death at 90 days) §	1 (4.8%)	2 (8.0%)	0.999

* Significant p values with FDR < 10% are noted with an asterisk.

† Adjusted p-value was computed using bootstrap linear regression model to evaluate mean differences between subgroups adjusting for treatment group, PRISM score and baseline use of steroids.

‡ Data presented as mean +/- SEM by bootstrapping

§ Adjusted p-value was computed using the Fisher’s exact test.

|| Data presented as median (interquartile range)

** Data presented as n (percentage)

**Table 3:** Differences in Inflammatory Biomarker Levels between Subclass 1 and 2

Biomarkers (pg/mL)	Subclass 1	Subclass 2	False Discovery Rate (FDR) Adjusted p-value†
PAI1	500 (224–887)	200 (94–308)	0.013*
IL4	7919 (3270–11897)	4510 (1770–10910)	0.32
IL6	148 (49–699)	15 (9–52)	<0.001*
IL8	34 (24–217)	14 (13–20)	<0.001*
IL10	13 (10–72)	10 (7–18)	0.027*
ANG2	6936 (3255–8587)	3414 (1901–6216)	0.035*
Thrombomodulin	4891 (4234–6531)	4144 (3389–5651)	0.15
TFPI	12027 (7262–18706)	13440 (7036–17308)	0.72
TREM1	489 (330–578)	325 (258–427)	0.044*
P-selectin	6414 (4880–8740)	6878 (4623–8885)	0.93
ICAM1	130000 (97558–193000)	113000 (80346–164000)	0.36
TNFR-1	1833 (1466–2284)	1106 (932–1296)	<0.001*

Data presented as median (IQR)

* Significant FDR values are noted with an asterisk.

† P-values computed by Wilcoxon rank sum test and adjusted by FDR criterion

## Data Availability

The datasets supporting the conclusions of this article are available from the corresponding author on reasonable request. JY, MSZ, and AS had full access to all the data in the study and take responsibility for the integrity of the data and the accuracy of the data analysis.

## Abbreviations

BCR: B-cell receptors
CAF-PINT: Coagulation and Fibrinolysis in Pediatric Insulin Titration Trial
DEG(s): Differentially expressed gene(s)
FDR: False Discovery Rate
GEDIT: Gene Expression Deconvolution Interactive Tool
GO: Gene Ontology
HALF-PINT: Heart and Lung Failure-Pediatric Insulin Titration Trial
(P)ICU: (Pediatric) Intensive Care Unit
KEGG: Kyoto Encyclopedia of Genes and Genomes
PELOD: Pediatric Logistic Organ Dysfunction
PRISM: Pediatric Risk of Mortality
RNA-Seq: Ribonucleic acid sequencing
TCR (A/ B/ D/ G): T-cell receptors (alpha/ beta/ delta/ gamma)
